# Exergaming Compared to Conventional Physical Exercise Interventions on Health Status in Older People with Parkinson’s Disease: A Systematic Review with Meta-Analysis of Randomized Controlled Trials

**DOI:** 10.3390/medicina61112001

**Published:** 2025-11-08

**Authors:** Jordan Hernandez-Martinez, Braulio Henrique Magnani Branco, Izham Cid-Calfucura, Tomás Herrera-Valenzuela, Nicole Fritz-Silva, Yeny Concha-Cisternas, Mauricio Barramuño-Medina, Edgar Vásquez-Carrasco, Joaquín Pérez-Cárcamo, Pablo Valdés-Badilla

**Affiliations:** 1 Department of Physical Activity Sciences, Universidad de Los Lagos, Osorno 5290000, Chile; jordan.hernandez@ulagos.cl (J.H.-M.); joaquinalejandro.perez@alumnos.ulagos.cl (J.P.-C.); 2 Department of Education, Faculty of Humanities, Universidad de la Serena, La Serena 1700000, Chile; 3 Graduate Program in Health Promotion, Cesumar University (UniCesumar), Maringá 87050-900, Brazil; braulio.branco@unicesumar.edu.br; 4 Escuela de Ciencias del Deporte y Actividad Física, Facultad de Salud, Universidad Santo Tomas (UST), Santiago 8370003, Chile; izham.cid@gmail.com; 5 Department of Physical Activity, Sports and Health Sciences, Faculty of Medical Sciences, Universidad de Santiago de Chile (USACH), Santiago 8370003, Chile; tomas.herrera@usach.cl; 6 Department of Health, Universidad de Los Lagos, Puerto Montt 5480000, Chile; nicole.fritz@ulagos.cl; 7 School of Kinesiology, Faculty of Health, Universidad Santo Tomás, Talca 3460000, Chile; yenyf.concha@gmail.com; 8 Vicerrectoría de Investigación e Innovación, Universidad Arturo Prat, Iquique 1100000, Chile; 9 Kinesiology Program, Faculty of Health Sciences, Universidad Autónoma de Chile, Temuco 7500912, Chile; mauricio.barramuno@uautonoma.cl; 10 Occupational Therapy School, Faculty of Psychology, University de Talca, Talca 3465548, Chile; edgar.vasquez@utalca.cl; 11 Centro de Investigación en Ciencias Cognitivas, Faculty of Psychology, Universidad de Talca, Talca 3465548, Chile; 12 VITALIS Longevity Center, Universidad de Talca, Talca 3465548, Chile; 13 Department of Physical Activity Sciences, Faculty of Education Sciences, Universidad Católica del Maule, Talca 3530000, Chile; 14 Sports Coach Career, Faculty of Life Sciences, Universidad Viña del Mar, Viña del Mar 2520000, Chile

**Keywords:** exergaming, virtual reality, neurologic disorders, aged, physical fitness

## Abstract

*Background and Objectives*: This systematic review aimed to analyze published peer-reviewed studies on the effects of exergaming (EXG) compared to conventional physical exercise (CPE) interventions on health status in older people with Parkinson’s disease (PD) according to training dose. *Materials and Methods*: Using six generic databases: PubMed, EBSCO, Medline, CINAHL Complete, Scopus, and Web of Science, the PRISMA, TESTEX, RoB 2, and GRADE tools assessed methodological quality and certainty. The protocol was registered in PROSPERO (code: CRD42024575969). *Results*: Out of 805 records, 14 randomized controlled trials with 406 older people with PD were included. Seven overall meta-analyses showed significant improvements (*p* < 0.01) in favor of EXG in the Berg Balance Scale (BBS, ES = 0.90), Dynamic Gait Index (DGI, ES = 0.77) and quality of life questionnaire (PDQ-39, ES = 0.52), without significant improvements (*p* > 0.05) in the Unified PD Rating Scale, Montreal Cognitive Assessment, Timed Up-and-Go and Falls Efficacy Scale-International. Four subgroup meta-analyses, according to training schedules, showed that there were significant improvements (*p* < 0.05) in BBS in favor of EXG at >8 weeks of training (ES = 1.38), >3 weeks per week (ES = 1.18), <45 min duration (ES = 0.99), and with >20 total sessions (ES = 1.31). Both weeks and total sessions were predictors of BBS performance in EXG interventions in older people with PD. *Conclusions*: EXG is an innovative alternative to improve the health status in balance, gait, and quality of life variables in older people with PD, with a high potential for clinical practice in this population. The training dose is a determinant (weeks and total sessions) that varies the response to intervention in the BBS.

## 1. Introduction

Parkinson’s disease (PD) is considered the second most common neurodegenerative disorder in the world [1]. Marked by degeneration of dopaminergic neurons in the substantia nigra pars compacta and the presence of Lewy bodies [2], as the pathology advances, additional regions such as the spinal cord, limbic system, nucleus accumbens, forebrain, and neocortex become compromised [2]. The main motor manifestations include tremor, rigidity, bradykinesia or akinesia, and postural instability, leading to slowness of movement, resting tremors, and impaired gait and posture [3]. These symptoms impact physical, motor, and cognitive function in middle-aged and older people [2,3], leading to impairments in memory [4], motor control [3], balance [5], gait [4], and muscle strength [6], which increase fall risk [7], affecting their functional independence [8], health-related quality of life (HRQoL), and general health status [8].

However, physical exercise interventions can help improve physical, motor, and cognitive function in older people with PD [9,10]. Mood, a key factor for enabling individuals with PD to carry out activities of daily, improved through physical exercise [11]. It also helps reduce the risk of dementia, which is highly prevalent in the advanced stages of PD [12]. In a meta-analysis of randomized controlled trials (RCTs) conducted by Ban et al. [13] in adults and older people with PD through yoga interventions, significant improvements were reported in motor function with the Unified Parkinson’s Disease Rating Scale (UPDRS-III test), balance was assessed through the Berg Balance Scale (BBS) and the Timed Up-and-Go (TUG) test, and health-related quality of life (HRQoL) was measured with the PDQ-39, in comparison with inactive control groups. However, a meta-analysis of RCTs in adults with PD did not report a significant improvement in the UPDRS-III test in favor of yoga interventions compared to inactive control groups [14]. On the contrary, in a meta-analysis of RCTs conducted by Johansson et al. [15] in older people with PD, motor training interventions combined with cognitive training reported significant improvements in gait speed but no improvements in TUG compared to active/inactive control groups. Similarly to that reported by Suárez-Iglesias et al. [16] in a meta-analysis of RCTs in older people with PD, Pilates interventions reported significant improvements in TUG compared to active/inactive control groups. In a meta-analysis of RCTs and non-randomized controlled trials (NRCTs) conducted by Radder et al. [9] on different physical exercise interventions (resistance training, treadmill training, aerobic exercises, martial arts, and dance), compared to inactive control groups, no significant improvements were reported in the Activities-specific Balance Confidence (ABC) scale for conventional physical exercise or TUG for dance training in older people with PD. However, significant differences were reported in favor of martial arts, balance training, hydrotherapy, exergaming (EXG), and conventional physical exercise (CPE) in the Dynamic Gait Index (DGI), BBS, TUG, Falls Efficacy Scale-International (FES-I), UPDRS-Part III and PDQ-39 compared to the control groups.

EXG could be an effective complementary physical exercise intervention for older people with neurodegenerative diseases, potentially improving physical function when used alongside usual care [17,18]. It may also enhance cognitive function [10,19], with such improvements contributing to a better HRQoL [20]. Studies conducted in healthy older people showed high activity enjoyment and adherence [21,22]; these results could be extrapolated to individuals with PD, given the key role of motivation in both groups. EXG has shown positive effects on brain structure and function, as well as on cognitive performance, by combining physical exercise and mental stimulation in interactive environments [23,24]. Recent studies have shown that this type of intervention promotes neuroplasticity by increasing neurotrophic factors and improving functional connectivity in frontoparietal and hippocampal networks [25]. A recent systematic review confirms that EXG modulated the brain regions involved in attention, motor coordination, and cognitive control, promoting structural and functional adaptations that were consistent with greater brain efficiency [26]. The EXG intervention consists of full-body movements that replicate real-life actions, performed in a limited space, with or without a controller to enable interaction with the virtual game environment [25,26], in virtual reality (VR), in a non-inversive way in front of a screen or in an immersive way with the use of glasses in VR [27]. In a meta-analysis with RCTs and NRCTs conducted by Zhang, Luximon, Pang, and Wang [18] on mobility and balance in older people with PD using non-immersive EXG interventions, significant improvements were reported in TUG, a 6 min walk test (6MWT), BBS, and DGI compared to CPE interventions. However, in a meta-analysis of RCTs conducted by Navarro-Lozano et al. [17] in older people with PD using non-immersive EXG interventions, no significant improvements in gait speed and BBS were reported compared to traditional visual and auditory interventions. However, this included five studies, which decreases the robustness of the results. Similar results to those reported in a meta-analysis of older people with PD on mobility, balance, and HRQoL with non-immersive EXG interventions showed significant improvements in PDQ-39, ABC scale, BBS, and DGI, but no significant differences in TUG and UPDRS-part II compared to CPE [20].

While there is evidence of the benefits of EXG on physical function and HRQoL in older people with PD compared to CPE interventions [17,18,20], in health and sports sciences, it is essential to focus on older people to update the evidence [28]. Furthermore, the synthesis of health status outcomes on variables such as the motor, physical, cognitive function and HRQoL of EXG according to training dosage compared to conventional interventions in individuals with PD remains unknown, as the methodological quality of existing studies has not yet been systematically evaluated. In this regard, this systematic review and meta-analysis sought to examine the existing peer-reviewed literature assessing the effects associated with EXG when contrasted with CPE interventions on physical function, cognitive function, balance, fall risk, and HRQoL in older people with PD, according to the training dosage.

## 2. Methods

### 2.1. Protocol and Registration

The present review was conducted in accordance with the PRISMA (Preferred Reporting Items for Systematic Reviews and Meta-Analyses) guidelines [29]. The protocol is registered with PROSPERO (the International Prospective Register of Systematic Reviews; ID code: CRD42024575969).

### 2.2. Eligibility Criteria

The inclusion criteria for this systematic review with meta-analysis were met by original, peer-reviewed articles published until September 2025 that were unrestricted by language or publication date. The materials that were excluded were conference abstracts, books and book chapters, editorials, letters to the editor, protocol records, reviews, case studies, and trials. In addition, a systematic review utilizing the PICOS framework, which stands for population, intervention, comparator, outcome, and study design, was included in the research (see Table 1).

### 2.3. Information and Database Search Process

The literature search was performed from December 2024 to September 2025 across six major databases: PubMed, Psychology and Behavioral Sciences Collection (EBSCO), Medline, CINAHL Complete, Scopus, and Web of Science (Core Collection). Both Medical Subject Headings (MeSH) from the U.S. National Library of Medicine and free-text terms were used to capture studies related to EXG, CPE, physical and cognitive function, balance, fall risk, and HRQoL in older adults with PD. The full search strategy for each database included keywords and Boolean operators used to search the databases: (“exergames” OR “exergaming” OR “active video games” OR “virtual reality” OR “non-immersive virtual reality” OR “immersive virtual reality” OR “wii” OR “Kinect” OR “playstation”) AND (“physiotherapy” OR “physical therapy” OR “physical exercise” OR “exercise” OR “traditional therapy” OR “balance training” OR “gait training”) AND (“physical function” OR “physical performance” OR “physical fitness” OR “functionality” OR “functional Independence” OR “functional dependency” OR “functional mobility” OR “health condition” OR “falls” OR “fall risk” OR “risk of fall” OR “falling risk” OR “balance” OR “static balance” OR “dynamic balance” OR “walking speed” OR “gait speed” OR “mobility”) AND (“cognition” OR “cognition functions” OR “executive functions” OR “executive control” OR “cognitive functioning” OR “cognitive control” OR “cognitive functions” OR “cognitive function” OR “memory” OR “cognitive abilities” OR “cognitive status” OR “global cognition” OR “mental flexibility” OR “memory functioning” OR “executive function” OR “short-term memory” OR “long-term memory” OR “cognitive” OR “neurocognition” OR “neurocognitive” OR “neuro-cognition” OR “neuro-cognitive” OR “executive functioning” OR “brain” OR “brain function” OR “brain structure” OR “brain development” OR “cognitive performance” OR “language”) AND (“QoL” OR “HRQoL” OR “quality of life” OR “quality of life perception” OR “health related quality of life” OR “health-related quality of life” OR “mental health” OR “psychological health” OR “body image perception” OR “life satisfaction” OR “lifestyle” OR “healthy lifestyle” OR “psychological well-being” OR “emotional well-being” OR “health status” OR “health status indicators” OR “vitality”) AND (“elderly” OR “older adults” OR “older people” OR “older subject” OR “aging” OR “aging” OR “aged”) AND (“Parkinson” OR “Parkinson’s disease” OR “neurological diseases”). To identify additional relevant studies, two independent experts were consulted who met the following criteria: (i) doctoral degree in sports science and (ii) peer-reviewed publications on physical performance across diverse populations or in journals indexed with an impact factor (Journal Citation Reports^®^). Their searches were conducted independently, without access to our strategy, to minimize bias. Finally, on 9 September 2025, a database check was performed to identify any retractions or errata for the included studies.

### 2.4. Study Selection and Data Collection Process

Studies were exported using the EndNote reference manager (version X9, Clarivate Analytics, Philadelphia, PA, USA). Two authors (JHM and ICC) independently screened the records, removed duplicates, and evaluated titles, abstracts, and full texts. No discrepancies were identified during this initial review. The same procedure was applied to studies suggested by external experts and to references cited within the included articles. Full texts of potentially eligible studies were then examined, and reasons for excluding studies that did not meet the selection criteria were documented.

### 2.5. Methodological Quality Assessment

The methodological quality of the included studies was evaluated using TESTEX, a scale specifically designed for exercise-based intervention studies [31]. TESTEX scores served as a potential criterion for exclusion [31]. The tool assigns a maximum of 15 points (5 for study quality and 10 for reporting), as outlined by Smart et al. [31]. Two authors (JHM, ICC) conducted the assessment independently, with a third author (THV) acting as an adjudicator for borderline cases, which were further reviewed and confirmed by an additional author (PVB).

### 2.6. Data Synthesis

From the included studies, the following information was extracted and analyzed: (i) author and publication year; (ii) country; (iii) study design; (iv) baseline health status of participants; (v) number of participants in intervention and control groups; (vi) mean age; (vii) activities performed in EXG and CPE; (viii) training volume (total duration, weekly frequency, and session length); (ix) training intensity; (x) instruments used to assess physical function, cognitive function, balance, fall risk, and HRQoL; and (xi) main outcomes reported.

### 2.7. Risk of Bias in Individual Studies

Two independent reviewers (JHM and ICC) assessed the risk of bias in the included studies using version 2 of the Cochrane Risk of Bias tool (RoB 2), with a third reviewer (PVB) resolving discrepancies. This evaluation followed the Cochrane Handbook for Systematic Reviews of Interventions [32] and considered randomization procedures, deviations from intended interventions, missing outcome data, outcome measurement, and the selection of reported results. Studies were then classified as having a “high,” “low,” or “some concerns” risk of bias.

### 2.8. Summary Measures for Meta-Analysis

The methodology of this study incorporated a meta-analysis, with full details registered in PROSPERO (registration code: CRD42024575969). Meta-analyses were conducted only when at least three studies were available [33]. Effect sizes (ES; Hedge’s g) were calculated for each outcome, including balance, mobility, fall risk, physical and cognitive function, HRQoL, body composition, physical performance, and biomarkers, using pre- and post-intervention means and standard deviations. Change score SDs were used to standardize data. ES values are reported with 95% confidence intervals (95% CIs) and interpreted according to the following scale: trivial < 0.2; small > 0.2–0.6; moderate > 0.6–1.2; large > 1.2–2.0; very large > 2.0–4.0; and extremely large > 4.0 [34].

A random-effects model was applied to account for inter-study variability potentially affecting EXG outcomes, using Comprehensive Meta-Analysis software (Version 2.0; Biostat, Englewood, NJ, USA). Statistical significance was set at *p* ≤ 0.05 [35]. For each trial, the DerSimonian-Laird random-effects approach was employed to calculate and pool SMD and MD for BBS, TUG, DGI, FES-I, UPDRS-Part III, MoCA, and PDQ-39 (EXG vs. CPE). This model assumes that true effects, influenced by factors such as intervention type and duration, vary across studies, reflecting populations with different effect sizes. Data were pooled only when at least three studies reported the same outcome [36].

Heterogeneity was assessed using Cochran’s Q test and the *I*^2^ statistic, with *I*^2^ values of <25%, 25–50%, and >50% indicating low, moderate, and high heterogeneity, respectively [37]. Egger regression tests were conducted to examine small-study effects and potential publication bias [38].

#### 2.8.1. Moderator Analysis

Potential sources of heterogeneity that could influence training effects were identified a priori using a random-effects model and independently computed single-factor analyses.

#### 2.8.2. Subgroup Analyses

Given that adaptive responses to EXG programs may be influenced by the training dose, including weeks of training, sessions per week, minutes per session, and total number of sessions [39], these variables were treated as potential moderators.

#### 2.8.3. Meta-Regression

A multivariate random-effects meta-regression was performed to determine whether training variables (frequency, duration, session length, and total sessions) predicted EXG effects on physical and cognitive function, balance, fall risk, and HRQoL. Each covariate analysis included a minimum of nine studies [40].

### 2.9. Certainty of Evidence

The certainty of evidence for each study was classified as high, moderate, low, or very low using the GRADE (Grading of Recommendations, Assessment, Development, and Evaluation) approach [41]. Since only experimental studies (RCTs) were included, all analyses initially assumed a high level of confidence, which was downgraded when concerns arose regarding the risk of bias, consistency, precision, directness of outcomes, or potential publication bias [41]. Two authors (JHM and ICC) performed the evaluations independently, with discrepancies resolved through discussion with a third author (PVB).

## 3. Results

### 3.1. Study Selection

**Figure 1:**
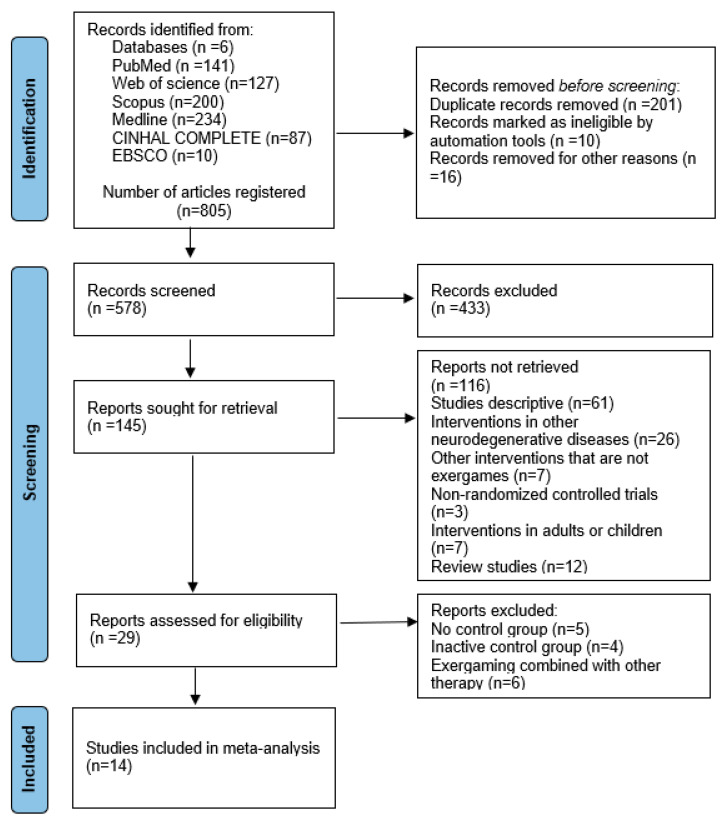
Flowchart of the review process. Legends: Based on the PRISMA guidelines [29].

Figure 1 presents the flow of the study selection. The initial search retrieved 805 records. After removing duplicates and screening titles, abstracts, and keywords, 578 records remained. Full-text assessment led to the exclusion of 433 articles that did not meet the inclusion criteria, leaving 145 studies. Additional exclusions included 61 descriptive studies, 26 interventions targeting other neurodegenerative diseases, seven non-EXG interventions, three non-randomized controlled trials, seven studies with participants outside the target age range, and 12 review articles. This resulted in 29 potentially eligible studies, from which five lacked a control group, four used inactive controls, and six combined EXG with other therapies. Ultimately, 14 studies met all inclusion criteria [42,43,44,45,46,47,48,49,50,51,52,53,54,55,56,57].

### 3.2. Methodological Quality

The 14 included studies were assessed using the TESTEX scale (Table 2). Scores ranged from 9/15 [30], with 10/15 [31], 11/15 [32,33,34,35,36], 12/15 [37,38,39,40,41,42,43,44], and 13/15 [45] indicating moderate-to-high methodological quality. As all studies scored at least 60%, none were excluded from the systematic review.

### 3.3. Risk of Bias Within Studies

**Figure 2:**
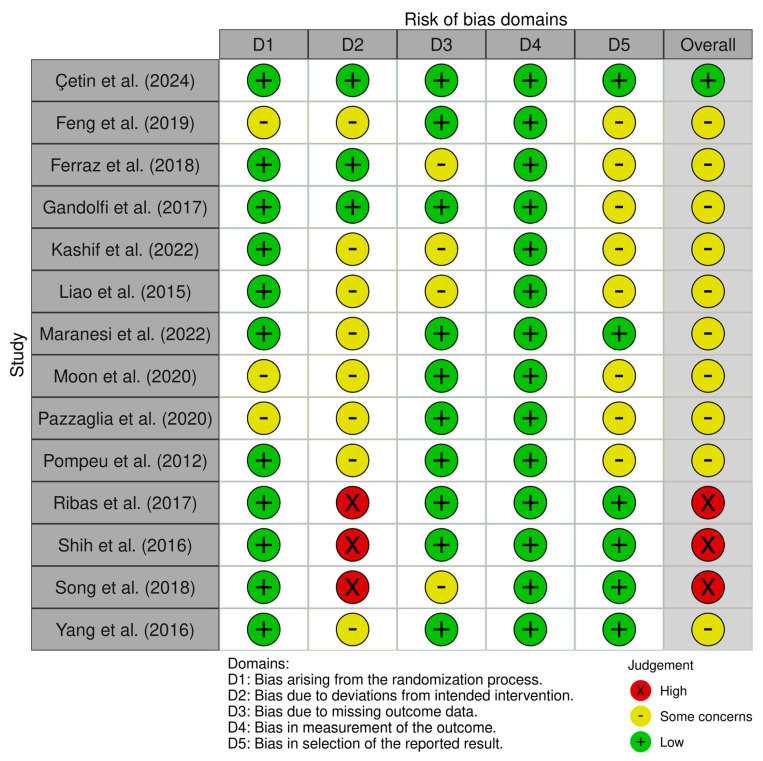
Risk of bias within studies [30,31,32,33,34,35,36,37,38,40,41,42,44,45]. D1: randomization process; D2: deviations from the intended interventions; D3: missing outcome data; D4: measurement of the outcome; and D5: selection of the reported result. Risk of bias assessment for included studies. Legend: D1, randomization process; D2, deviations from intended interventions; D3, missing outcome data; D4, outcome measurement; and D5, selection of reported results.

**Figure 3:**
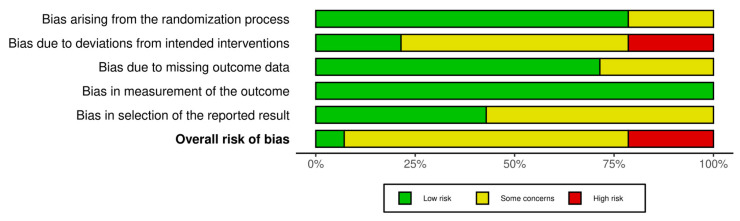
Overview of the authors’ evaluations for each bias domain across all included studies.

One study was assessed as having a low risk of bias across all domains [31]. Ten studies showed some concerns in one or more domains [30,32,33,34,37,38,39,40,42,43,44,45]. Three studies were classified as having a high risk of bias [35,36,41]. Overall, the risk of bias was moderate, with most studies presenting some concerns and only a few demonstrating low risk across all domains. Figure 2 and Figure 3 present the risk of bias.

### 3.4. Studies Characteristics

Nine studies were developed in Europe [31,32,33,34,35,36,37,38,45], three in Asia [30,40,44], and two in South America [41,42]. Fourteen studies of RCTs presented a total of 406 participants with stage 1 to 3 Parkinson’s disease with a mean age of 73 years. The duration of the interventions ranged from 4 to 15 weeks; with two to five weekly sessions of 15 to 90 min, only one study reported the intensity [37], with moderate-to-high intensities ranging from 12 to 15 on the 20-point rating of perceived exertion (RPE) and 50% to 75% maximum heart rate (HRmax). Non-immersive VR was used in the experimental groups, through EXG with consoles such as Xbox Kinect and Nintendo Wii for sports and adventure games. Regarding CPE interventions, balance training, gait training, and balance training combined with gait were used. All the results of the 14 studies analyzed are presented in detail in Table 3.

### 3.5. Meta-Analysis Results

The overall effects of EXG on balance, mobility and fall risk, physical and cognitive function, and HRQoL variables are shown in Table 4. There were moderate-to-large significant effects (*p* < 0.05) in favor of EXG in BBS, DGI, and PDQ-39 (ES = 0.52 to 0.90). However, there were no significant effects (*p* > 0.05) on TUG, FES-1, UPDRS-Part III, and MOCA (ES = −0.21 to 1.57).

### 3.6. Meta-Analysis Subgroup

#### Subgroup Analysis by Dosage Training

**Figure 4:**
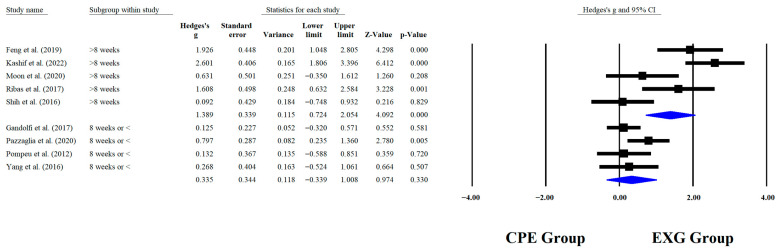
Forest plot of changes in BBS in older people with Parkinson’s disease participating in exergaming training compared with older people with obesity and Parkinson’s disease, assigned with conventional physical exercises with weeks of training [30,32,34,35,38,41,42,44,45]. Values shown are effect sizes (Hedges’ g) with 95% confidence intervals (CI). CPE: conventional physical exercise; EXG: exergaming.

**Figure 5:**
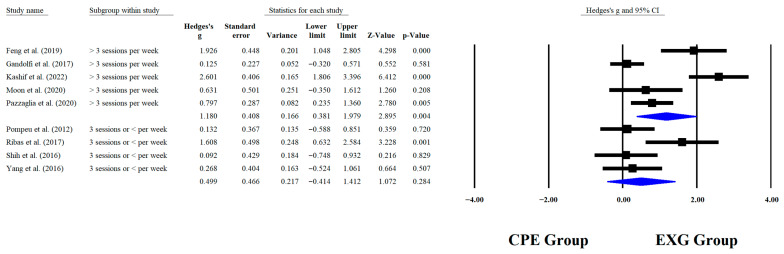
Forest plot of changes in BBS in older people with Parkinson’s disease participating in exergaming training compared with older people with obesity and Parkinson’s disease, assigned as conventional physical exercises with frequency of training [30,32,34,35,38,41,42,44,45]. Values shown are effect sizes (Hedges’ g) with 95% confidence intervals (CI). CPE: conventional physical exercise; EXG: exergaming.

**Figure 6:**
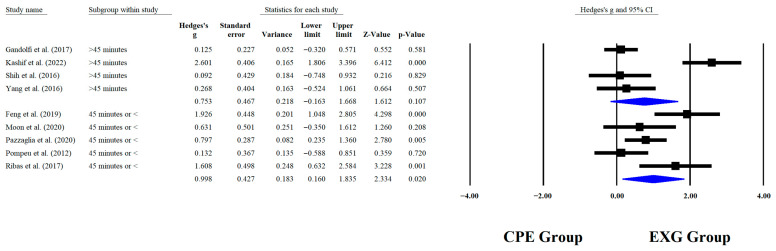
Forest plot of changes in BBS in older people with Parkinson’s disease participating in exergaming training compared with older people with obesity Parkinson’s disease assigned as conventional physical exercises with minutes per sessions [30,32,34,35,38,41,42,44,45]. Values shown are effect sizes (Hedges’ g) with 95% confidence intervals (CI). CPE: conventional physical exercise; EXG: exergaming.

**Figure 7:**
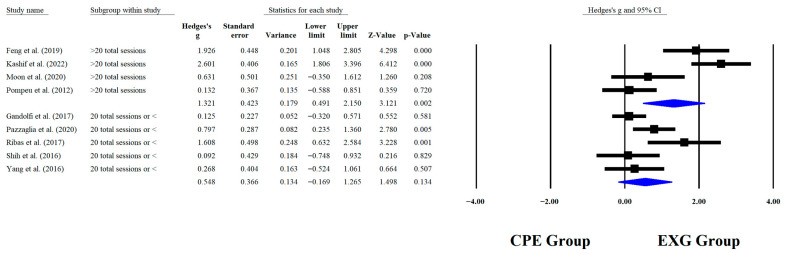
Forest plot of changes in BBS in older people with Parkinson’s disease participating in exergaming training compared with older people with obesity and Parkinson’s disease, assigned as conventional physical exercises with total sessions [30,32,34,35,38,41,42,44,45]. Values shown are effect sizes (Hedges’ g) with 95% confidence intervals (CI). CPE: conventional physical exercise; EXG: exergaming.

Regarding the training dose in the balance test with BBS, there were significant improvements (*p* < 0.05) in favor of EXG with interventions >8 weeks with a total of >20 sessions and <45 min per session, with very long effects (ES = 0.99 to 1.38). However, in the training frequency, no significant improvements (*p* > 0.05) were reported in favor of EXG with moderate-to-large effects (ES = 0.49 to 1.18). These results are presented graphically in Figure 4, Figure 5, Figure 6 and Figure 7.

### 3.7. Meta-Regression

The calculation of the meta-regression was performed with at least nine studies per covariate. BBS was taken into account for the meta-regression analysis, which analyzed four training variables (weeks, frequency, minutes per session, and total number of sessions) (Table 5). In the BBS in the weeks and total sessions of intervention, BBS was found to be a predictor of the effect of EXG in the aforementioned test (*p* < 0.05).

### 3.8. Adverse Effects and Adherence

Only one study presented adverse effects such as nausea, dizziness, and vertigo [43,44]. All studies [30,31,32,33,34,35,36,37,38,40,41,42,43,44,45,46,47,48] achieved adherence equal to or higher than 80% in the EXG interventions. Eight studies reported that the interventions were supervised by certified physiotherapists [31,33,34,36,37,38,41,42,43,44,47,48], two studies had their interventions supervised by certified occupational therapists [32,45,46], and three studies did not report supervision in their interventions [30,35,40]. Only one study reported pre-intervention familiarization sessions [43,44].

### 3.9. Certainty of Evidence

The analysis of the available RCTs suggests that the evaluated intervention may have a moderate beneficial effect on various health domains in individuals with PD, including balance, mobility, and physical and cognitive function, as well as HRQoL. However, the certainty of evidence is considered moderate due to the presence of identified risks of bias in the included studies. Therefore, these findings should be interpreted with caution, and further research is recommended to confirm the results and strengthen the existing evidence base (Table 6).

## 4. Discussion

In this systematic review with meta-analysis, the main results in favor of EXG interventions are significant improvements in static balance (BBS), mobility (DGI), and HRQoL (PDQ-39) compared to CPE in older people with stage 1 to 3 PD.

### 4.1. UPDRS-Part III

The present meta-analysis shows significant improvements favoring EXG vs. CPE in UPDRS-Part III. These results are similar to those reported by by Rodríguez-Mansilla et al. [49] in a systematic review of RCTs and NRCTs in older people with PD, presenting improvements in UPDRS-Part III through interventions with Nintendo Wii Fit and Xbox Kinect Adventure compared to active/inactive control groups. Similarly, Kashif et al. [50], in a systematic review of RCTs and NRCTs in adults and older people with PD, presented improvements in UPDRS-Part III through interventions with Nintendo Wii Fit and VR training compared to CPE.

The results of our meta-analysis confirm the existing findings in the literature. At the same time, CPE provides a specific program of active exercises that challenge the center of pressure in combination with aerobic balance exercises [20,51]; visual stimuli through EXG helps to adjust the alignment of the limbs during the games and to determine the direction of movement through a combination of visual and sensory information [52]. In this sense, Pompeu et al. [53] have mentioned that the main factor that generated improvements in the learning of various motor functions is the constant visual and auditory feedback provided by the EXG. The sensory feedback associated with virtual exercises seems to activate the mirror neuron systems of the central nervous system, which would be able to store a memory of the representation of the movement performed in the primary motor cortex, dorsal premotor cortex, and supplementary motor cortical areas [49,54], allowing for more significant improvements in motor learning and task performance compared to CPE in people with PD [49,55].

### 4.2. Montreal Cognitive Assessment (MOCA)

The present meta-analysis shows significant improvements in favor of EXG in cognitive function compared to CPE. Similar results were observed in a systematic review by Garcia-Agundez et al. [10], which analyzed RCTs and pilot studies involving older people with PD. The review highlighted significant improvements in cognitive function, as measured by the MOCA, when using interventions such as Nintendo Wii Fit (Nintendo Co., Ltd., Kyoto, Japan) and Xbox Kinect (Microsoft Corporation, Redmond, WA, USA), compared to active/inactive control groups. Similarly, in a systematic review using Nintendo Wii Fit, Barry et al. (2014) [19] showed improvements in cognitive function using MOCA in older people with PD compared to CPE with balance and gait training. As previously mentioned, VR gaming is a technology that allows information to flow in and out of the visual system. Motor actions are displayed in the game’s virtual environment, and the system provides multimodal feedback related to the movement’s execution [49]. Through external and internal senses, sensory feedback is integrated into the patient’s mental representation [56]. VR has been shown to improve cognitive and motor skills, such as attention and executive function, with positive responses in physical symptoms such as the freezing of gait [10]. In a study by Mendes et al. [57], who aimed to assess learning, retention, and transfer of performance improvements after Nintendo Wii Fit training in PD patients, they assessed motor and cognitive gameplay ability, showing that PD participants failed to improve in games that required decision making and quick movements to avoid virtual obstacles. During EXG training, the integration of motor tasks with cognitive demands, such as decision making, working memory, or inhibitory control, induces greater functional neuroplasticity in the fronto-striatal and parietal circuits, which are especially vulnerable in this disease [58,59]. This finding is important, given that introducing cognitively demanding aspects slowly and moderately seems to be key to inducing improvements in cognitive skills (MOCA scores) [19]. Furthermore, when playing games that are too fast or complex, motivation, adherence, and safety may be compromised [19].

### 4.3. PDQ-39

Another result reported in the present meta-analysis was a significant improvement in HRQoL by PDQ-39 in favor of EXG vs. CPE. Similar results to those reported by Elena et al. [20] in a meta-analysis of older people with PD showed significant improvements in favor of EXG (*p* < 0.001) in PDQ-39 compared to conventional physiotherapy. Similarly, in an overview conducted by Rocha et al. [60] in older people with PD, significant improvements were reported in favor of EXG vs. inactive control groups in PDQ-39 (*p* < 0.05) and EXG combined with CPE vs. only CPE (*p* < 0.01). When patients are diagnosed with PD, it has been reported that their dopamine levels decrease by approximately 70% in the nigrostriatal pathway; this can favor conditions such as depression, which occurs in many patients, causing problems in carrying out their activities of daily living, affecting their HRQoL [61]. However, EXG programs have been shown to improve mental health by reducing stress and depression [62]. For example, Herz et al. [61] showed that significant improvements were achieved over 4 weeks with EXG for HRQoL, assessed by the PDQ-39, in activities of daily living, emotion, communication, bodily discomfort, and total score. In this sense, research has mentioned an increase in dopamine during participation in video games, which, together with the motor actions performed through EXG, can explain the findings of PDQ-39 [61].

### 4.4. BBS

Another result obtained in the present meta-analysis was a significant improvement in favor of EXG in the BBS compared to CPE, similarly to that reported by Zhang et al. [18] in a meta-analysis of older people with PD showing significant improvements (*p* < 0.001) in BBS in favor of interventions with Nintendo Wii Fit and Xbox Kinect compared to CPE. Similarly, in a meta-analysis of older people with PD, Elena et al. (2021) [20] showed significant improvements (*p* = 0.001) in BBS in favor of EXG compared to conventional physiotherapy. People with PD may present a range of neurological disorders associated with balance impairments [63]. Balance impairments can be reduced or compensated for by physical activities, involving postural control training and time-reaction practice [64]. In this sense, EXG involves constant self-correction, where users interact with different game scenarios. This interaction and action-observation of the avatar or character’s movements challenge users’ sensory perception [40]. Jorgensen et al. [65] have reported that older people need to control their center of pressure in multiple directions during EXG. Therefore, favorable results for EXG regarding balance may represent sensitivity in integrating the sensory modalities (vestibular, proprioceptive, auditory, and visual systems) necessary for balance when interacting with game scenarios [66].

### 4.5. TUG and DGI

Regarding TUG, the present meta-analysis showed significant improvements in dynamic balance by TUG in favor of EXG compared to CPE, with similar results to those reported by Zhang et al. [18] in a meta-analysis showing significant improvements (*p* = 0.01) in TUG in favor of EXG compared to CPE in older people with PD. Similarly, Sarasso et al. [67], in a meta-analysis conducted in older people with PD, showed significant improvements (*p* = 0.04) in favor of immersive and non-immersive VR interventions compared to balance training. Finally, no significant differences were reported for EXG and CPE in the DGI in the present meta-analysis, with similar results to those reported by Chen et al. [68] in a meta-analysis in older people with PD where no significant differences (*p* = 0.78) in the DGI were reported between interventions with VR vs. CPE. However, a meta-analysis by Elena et al. [20] reported significant improvements in DGI (*p* = 0.005) in favor of EXG compared to conventional physiotherapy in older people with PD.

TUG is used to assess fall risk in older people [69], and the DGI is used to assess balance and fall risk in older people [70]. Our meta-analysis reported significant improvements for the ABC scale and the TUG test; this can be attributed to better use of different resources for stability and postural orientation [62]. As mentioned above, balance deficiencies for activities of daily living can be compensated for by performing physical activities that involve postural control training, time-reaction practice, and reactive recovery [64]. In this sense, EXG has large degrees of freedom applied in different directions during video games, causing participants to change their center of pressure constantly; this continuously demands motor control from users. In addition, visual and auditory feedback, together with the gradual increase in difficulty in the games, implies greater participation of the sensorimotor system, which, unlike CPE, incorporates all the afferent components, the integration process, and the efferent responses to maintain functional joint stability during body movements [71,72]. Although these findings can also be extrapolated to the DGI, our meta-analysis did not report significant improvements, which can be attributed to the high heterogeneity in the analysis of the outcome measures [34,38,45,48], in addition to the different EXG used in each intervention, given that the difficulty of the EXG can play an essential role in adherence and balance improvements in older people, so the selection and design of the games is an important factor to consider [73].

### 4.6. FES-I

Another result obtained in the present systematic review with meta-analysis was that EXG did not improve the fall risk on the FES-I, similarly to what was reported in a systematic review of older people with PD by Mylonas et al. [74], wherein VR interventions presented no improvement in FES-I compared to active/inactive control groups. However, a systematic review conducted by García-López et al. [75] in older people with PD reported improvements in reducing the number of falls using non-immersive VR interventions compared to active/inactive control groups. It is significant to mention that FES-I does not represent the actual incidence of falls, but only assesses the worry and fear of falling, with questions limited to basic daily household activities and social activities outside the home [46]. After an EXG intervention, patients could have greater confidence in their daily tasks that require balance, such as walking on uneven surfaces or going up and down stairs [40]. However, Song et al. [36] reported that patients, despite increasing their confidence regarding their mobility, did not improve on the FES-I scale. This fact reflects that additional follow-up may be necessary after interventions in patients with PD, highlighting the importance of assessing individuals’ perceptions of the efficacy of interventions [40,46].

### 4.7. Subgroup Analysis According to Training Duration Weeks

When analyzing subgroups that were based on training duration, significant improvements were observed in BBS performance among older people with PD who participated in EXG programs with more than 8 weeks of sessions in total (*p* < 0.0001; ES = 1.38), whereas no significant effects were found in those who received 8 weeks of sessions or fewer (*p* = 0.330; ES = 0.33). Similar results were reported in RCTs of older people with PD, where the experimental group that received sessions for 8 weeks showed a significant improvement in balance as measured by the BBS, with a *p* < 0.004 [76]. Different results were reported in an RCT where app-guided training led to significant improvements in BBS (*p* < 0.0001), after completing 4 weeks in total [77]. In individuals with PD, dopaminergic dysfunction slows down adaptive and neuroplastic processes, making longer interventions (>8 weeks) more effective in promoting motor learning consolidation and postural control automation [78]. This effect is more evident when feedback-based approaches such as EXG are used, especially when combined with higher intensity, which further enhances the outcome.

### 4.8. Subgroup Analysis According to Training Frequency

When analyzing subgroups based on weekly training frequency, significant improvements were observed in BBS performance among older people with PD who engaged in EXG more than three times per week (*p* = 0.004; ES = 1.18), while no significant effects were detected in those who trained three times per week or less (*p* = 0.284; ES = 0.50). In another RCT, app-guided training led to significant improvements in BBS (*p* < 0.0001) after completing five sessions per week [77]. Similar results were reported in an RCT of older people with PD, where the experimental group received treadmill training combined with task-oriented circuit training three times per week for 8 weeks. The experimental group showed a significant improvement in balance as measured by the BBS, with a *p* < 0.004 [77]. Training more than three times per week can increase the repetition and intensity of motor stimuli, promoting long-term potentiation in motor and prefrontal circuits, which is key for cortical reorganization and the automation of postural control [79]. This highlights the need for more frequent weekly sessions to induce lasting functional changes through consistent neural stimulation.

### 4.9. Subgroup Analysis According to Minutes per Session

When analyzing subgroups based on session duration, significant improvements in BBS performance were observed for sessions lasting 45 min or less (*p* = 0.020; ES = 0.99). In contrast, no significant improvements were found for sessions longer than 45 min (*p* = 0.107; ES = 0.75). Different results were reported in an RCT of the effects of a 12-month intervention of Tai Chi on people with PD; only the Tai Chi group that trained for 60 min per session showed significant improvements in balance as measured by the BBS (*p* = 0.002) [80]. In another RCT involving individuals with PD, the experimental group performed dual-task aquatic exercises for 60 min per session. This group showed significant improvements in balance, as measured by the BBS, compared to the control group (*p* = 0.002) [81]. The optimal session duration may depend on exercise type, intensity, and participant capacity, with moderate, targeted sessions maximizing benefits without fatigue, while well-adapted longer interventions [80,81] can also be effective by providing sustained multidimensional stimulation.

### 4.10. Subgroup Analysis According to Total Sessions

When analyzing subgroups based on the total number of training sessions, significant improvements in outcomes were observed among participants who engaged in more than 20 sessions of EXG (*p* < 0.001; ES = 1.32–2.60). In contrast, studies involving 20 or fewer sessions did not demonstrate consistent effects, with most showing non-significant results (*p* > 0.05; ES = 0.09–0.80). Similar results were reported in an RCT where the experimental group performed aquatic dual task exercises over a total of 20 sessions, showing significant improvements both at the end of the intervention and after the detraining period (*p* = 0.002) [81]. Similar results were reported in an RCT where app-guided training led to significant improvements in BBS (*p* < 0.0001) after completing 20 total sessions [77]. Exceeding 20 sessions distributed over time induces long-term potentiation in the synapses of the motor and postural control circuits, reaching the threshold for stable structural changes in the motor cortex, cerebellum, and basal ganglia [79,80]. Distributed practice enhances motor retention by reducing interference from forgetting, thereby promoting the automation of balance strategies [78]. Overall, this session threshold optimizes both synaptic plasticity and the consolidation of motor learning.

### 4.11. Meta-Regression

Meta-regression analysis was conducted using at least nine studies per covariate, focusing on BBS outcomes and four training variables: duration in weeks, frequency, minutes per session, and total number of sessions. Significant predictors of EXG effects on BBS were the intervention duration (weeks, *p* < 0.01) and total number of sessions (*p* = 0.01), with coefficients of 0.21 and 0.03, respectively. Frequency and session duration were not significant predictors (*p* > 0.05). The highest explanatory power was observed for weeks (R^2^ = 0.80), followed by total sessions (R^2^ = 0.49). Therefore, these results support the importance of applying an adequate dose of intervention to improve balance in older people, which can have a positive impact on reducing their fall risk [82,83].

### 4.12. Strengths and Limitations

Among the limitations of this review are the following: (i) variability in the gaming consoles and software used, which may produce differing responses to EXG interventions; (ii) inconsistencies in comparisons, with some studies evaluating CPE versus EXG combined with CPE, and others comparing EXG alone to CPE; and (iii) limited analysis of neurophysiological responses between EXG and CPE interventions. The strengths include the following: (i) methodological quality above 60% in all included studies; (ii) adherence to established methodological frameworks, such as PRISMA, PROSPERO, TESTEX, RoB 2, and GRADE; (iii) use of six major databases—PubMed, EBSCO, Medline, CINAHL Complete, Scopus, and Web of Science; and (iv) inclusion of older adults with PD across Hoehn and Yahr stages 1 to 3. The findings of this systematic review with meta-analysis provide valuable insights for the rehabilitation of older adults with PD. Nevertheless, the long-term sustainability of these effects remains uncertain, highlighting the need for future studies incorporating extended follow-up assessments to evaluate the durability of EXG-based interventions.

### 4.13. Practical Applications

Based on the findings of this meta-analysis, EXG programs in older people with PD should exceed 8 weeks to allow for necessary neuroplastic adaptation [78] and should comprise more than 20 sessions to induce long-term potentiation in motor and postural control circuits [79,80]. It is recommended to schedule over three sessions per week to maximize repetition and stimulus intensity, promoting cortical reorganization and the automation of postural control [79]. Sessions of 30 to 45 min optimize focus and minimize neuromuscular fatigue, whereas 60 min sessions can be reserved for low-impact, individualized modalities [80,81]. Moreover, these guidelines support cognitive gains reflected in the increased MOCA scores [10] and the improved HRQoL, as evidenced by PDQ-39 reductions after four weeks of EXG [61,62]. Finally, progressively adjusting difficulty sustains motivation and safety, and periodic assessments enable monitoring of mid- and long-term effects, allowing protocols to be tailored to everyone’s response.

## 5. Conclusions

EXG showed significant improvements in BBS, DGI, and PDQ-39 compared to CPE in older people with PD. In addition, subgroup analysis showed significant improvements in BBS for a duration > 8 weeks with a frequency > 3 sessions per week < 45 min duration per session, for a total > 20 sessions. The predictors of BBS performance were weeks and total number of EXG intervention sessions. Future research could include supervised interventions, starting from the earliest stages of PD (1 and 2). Despite some limitations in the current evidence, there is sufficient support to recommend EXG for individuals with stage 1 to 3 PD, with strong benefits observed. Further studies should also explore the long-term adherence and sustainability of EXG interventions in real-world settings, as well as their impact on falls prevention, HRQoL, and functional independence.

## Figures and Tables

**Table 1:** Selection criteria used in the systematic review.

Category	Inclusion Criteria	Exclusion Criteria
Population	Older people defined by the World Health Organization as individuals with a mean age of 60 years or above [30], of either sex, diagnosed with PD. The severity of PD was classified according to the Hoehn and Yahr scale, stages 1 to 3.	Persons younger than 60 years with PD, those older than 60 without PD, patients in advanced disease stages (Hoehn and Yahr 4–5), people diagnosed with PD and other neurological diseases (e.g., dementia, Alzheimer’s disease, or moderate or advanced cognitive impairment).
Intervention	Interventions use EXG or active immersive and non-immersive video games (i.e., Wii sports, Balance and Fit, Switch Sports, Kinect Sports, Adventure and Your Shape, Sports Champions Move, NIRVANA) for 4 weeks or more.	Interventions that either did not involve EXG or combined EXG with other forms of physical therapy, and for which the intervention protocol was not clearly described.
Comparator	Interventions with active control groups receiving CPE-based programs (i.e., physiotherapy, balance training, gait training) through supervision of older people with Parkinson’s disease.	Studies without a control condition or with controls receiving no active intervention.
Outcome	At least one assessment of physical function (i.e., UPDRS-part II and UPDRS-part III), such as cognitive function, i.e., Montreal Cognitive Assessment (MOCA), Mini-Mental State Examination (MMSE), and balance and fall risk, including measures of static and dynamic stability, as well as gait and walking speed, or health-related quality of life (i.e., PDQ-39), before and after.	Lack of baseline data and/or follow-ups.
Study design	Randomized trials incorporating pre and post intervention evaluations	Non-randomized studies, including controlled trials, cross-sectional, retrospective, and prospective designs.

PD: Parkinson’s disease. EXG: exergaming. CPE: conventional physical exercise. MOCA: Montreal Cognitive Assessment. UPDRS-part II: Unified Parkinson’s Disease Rating Scale two. UPDRS-part III: Unified Parkinson’s Disease Rating Scale three. PDQ-39: quality of life questionnaire for PD.

**Table 2:** Evaluation of methodological rigor according to TESTEX criteria.

Study	Eligibility Criteria Specified	Randomly Allocated Participants	Allocation Concealed	Groups Similar at Baseline	Assessors Blinded	Outcome Measures Assessed >85% of Participants *	Intention to Treat Analysis	Reporting of Between Group Statistical Comparisons	Point Measures and Measures of Variability Reported **	Activity Monitoring in Control Group	Relative Exercise Intensity Reviewed	Exercise Volume and Energy Expended	Overall TESTEX #
Çetin, Kılınç and Çakmaklı [31]	Yes	Yes	Yes	Yes	No	Yes (1)	No	Yes	Yes (2)	Yes	No	Yes	10/15
Feng et al. [32]	Yes	Yes	Yes	Yes	Yes	Yes (1)	No	Yes	Yes (2)	Yes	No	Yes	11/15
Ferraz et al. [37]	Yes	Yes	Yes	Yes	Yes	Yes (1)	No	Yes	Yes (2)	Yes	Yes	Yes	12/15
Gandolfi et al. [38]	Yes	Yes	Yes	Yes	Yes	Yes (2)	No	Yes	Yes (2)	Yes	No	Yes	12/15
Kashif. et al. [44]	Yes	Yes	Yes	Yes	Yes	Yes (2)	No	Yes	Yes (2)	Yes	Unclear	Yes	12/15
Liao et al. [40]	Yes	Yes	Unclear	Yes	Yes	Yes (2)	Yes	Yes	Yes (2)	Yes	Unclear	Yes	12/15
Maranesi et al. [33]	Yes	Yes	Yes	Yes	Yes	Yes (1)	No	Yes	Yes (2)	Yes	No	Yes	11/15
Moon, Jung and Cho [30]	Yes	Yes	Unclear	Yes	No	Yes (1)	No	Yes	Yes (2)	Yes	No	Yes	9/15
Pazzaglia et al. [34]	Yes	Yes	Yes	Yes	Yes	Yes (1)	No	Yes	Yes (2)	Yes	No	Yes	11/15
Pompeu et al. [42]	Yes	Yes	Yes	Yes	Yes	Yes (2)	No	Yes	Yes (2)	Yes	No	Yes	12/15
Ribas et al. [41]	Yes	Yes	Yes	Yes	Yes	Yes (2)	No	Yes	Yes (2)	Yes	No	Yes	12/15
Shih et al. [35]	Yes	Yes	Yes	Yes	Yes	Yes (1)	No	Yes	Yes (2)	Yes	Unclear	Yes	11/15
Song et al. [36]	Yes	Yes	Yes	Yes	Yes	Yes (2)	No	Yes	Yes (2)	No	No	Yes	11/15
Yang et al. [45]	Yes	Yes	Yes	Yes	Yes	Yes (2)	Yes	Yes	Yes (2)	Yes	Unclear	Yes	13/15

* Three points can be awarded: one for adherence > 85%, one for reporting adverse events, and one for documenting exercise attendance. ** Two points can be assigned: one if the primary outcome is reported, and one if all additional outcomes are reported. Maximum score: 15 points. TESTEX #: tool for evaluating study quality and reporting in exercise interventions [31].

**Table 3:** Studies report the exergaming vs. conventional physical exercise intervention on physical function, cognitive function, balance, fall risk, and health-related quality of life in older people with Parkinson’s disease.

Study	Country	Study Design	Sample’s Initial	Groups	Mean Age (Years)	Type of Intervention and Control Group	Training Volume			Training Intensity	Balance and Fall Risk (Assessments)	Cognitive Function (Assessment)	Physical Function (Assessments)	HRQoL (Assessments)	Main Outcomes
				(n)			Weeks	Frequency (Sessions /Week)	Session Duration (min)						
Çetin, Kılınç and Çakmaklı [31]	UK	RCT	Subjects diagnosed with Parkinson’s Stage 2 through 3	EXG: 10 (70% male and 30% female) CPE: 10 (60% male and 40% female)	EXG: 68.5 (54–73) CPE: 70 (51–84)	EXG: Exergames Program CPE: Conventional balance training	8	3	60	NR	NR	MOCA (pt)	NR	NR	EXG vs. CPE EXG ↑ MOCA
Feng et al. [32]	UK	RCT	Subjects diagnosed with Parkinson’s Stage 2 through 3	EXG: 14 (53.33% male and 46.67% female) CPE: 14 (60% male and 40% female)	EXG: 67.47 ± 4.79 CPE: 66.93 ± 4.64	EXG: Virtual game balance CPE: Convectional gait training	12	5	45	NR	BBS (pt) TUG (s) Functional Gait Assessment (pt)	NR	UPDRS-part III (pt)	NR	EXG vs. CPE Both groups ↑ BBS ↑ TUG ↑ Functional Gait Assessment ↑ UPDRS-part III
Ferraz et al. [37]	UK	RCT	Subjects diagnosed with Parkinson’s Stage 2 through 3	EXG: 20 (50% male and 50% female) CPE1: 22 (72.73% male and 27.27% female) CPE2: 20 (55% male and 45% female)	EXG: 67 (66–68) CPE1: 71 (66–75) CPE2: 67 (64–71)	EXG: *Xbox Kinect Adventures* CPE1: Conventional balance training CPE2: Cycling static training	8	3	50	EXG: 15 RPE CPE1: 15 RPE. CPE2: 50% to 75% HRmax	NR	NR	NR	PDQ-39 (pt)	EXG vs. CPE1 vs. CPE2 EXG ↑ PDQ-39
Gandolfi et al. [38]	Italy	RCT	Subjects diagnosed with Parkinson’s Stage 2 through 3	EXG: 38 (60.53% male and 39.47% female) CPE: 38 (73.68% male and 26.32% female)	EXG: 67.45 ± 7.18 CPE: 69.84 ± 9.41	EXG: Nintendo Wii adventure CPE: Conventional balance training	4	5	50	NR	BBS (pt) ABC Scale (pt) DGI (pt)	NR	NR	PDQ-8 (pt)	EXG vs. CPE Both groups ↑ BBS ↑ ABC Scale ↑ DGI ↑ PDQ-8
Kashif. et al. [44]	Pakistan	RCT	Subjects diagnosed with Parkinson’s Stage 2 through 3	EXG: 20 (60% male and 40% female) CPE: 21 (52.38% male and 47.62% female)	EXG: 63.86 ± 4.57 CPE: 62.32 ± 4.61	EXG: Nintendo *Wii Sports* CPE: Physical therapy	12	3	60	NR	BBS (pt) ABC Scale (pt)	NR	UPDRS-part III (pt) UPDRS-part II (pt)	NR	EXG vs. CPE Both groups ↑ ABC Scale **EXG** ↑ BBS ↑ UPDRS-part III ↑ UPDRS-part II
Liao et al. [40]	Taiwan	RCT	Subjects diagnosed with Parkinson’s Stage 1 through 3	EXG: 12 (50% male and 50% female) CPE1: 12 (50% male and 50% female) CPE2: 12 (41.67% male and 58.33% female)	EXG: 67.3 ± 7.1 CPE1: 65.1 ± 6.7 CPE2: 64.6 ± 8.6	EXG: Nintendo Wii Fit CPE1: Conventional balance training. CPE2: Conventional gait training	6	2	60	NR	TUG (s) FES-I (pt)	NR	NR	PDQ-39 (pt)	EXG vs. CPE1 vs. CPE2 Only EXG and CPE2 ↑ TUG ↑ FES-1 ↑ PDQ-39
Maranesi et al. [33]	Italy	RCT	Subjects diagnosed with Parkinson’s Stage 1 through 3	EXG: 16 (37.50% male and 62.50% female) CPE: 14 (64.2% male and 35.8% female)	EXG: 72.7 ± 6.3 CPE: 75.5 ± 5.4	EXG: Virtual game *Apple Picking*; and game *The Labyrinth* CPE: Conventional balance training.	5	2	50	NR	POMA (pt) FES-I (pt) Gait Speed (m/s)	NR	NR	NR	EXG vs. CPE Both groups ↔ FES-I ↔ Gait Speed EXG ↑ POMA
Moon, Jung and Cho [30]	Republic of Korea	RCT	Subjects diagnosed with Parkinson’s Stage 2 through 3	EXG: 8 (62.5% male and 37.5% female) CPE: 7 (71.4% male and 28.6% female)	EXG: 63.38 ± 5.37 CPE: 62.14 ± 5.55	EXG: Nintendo Wii Fit CPE: Conventional balance and gait training	8	3	30	NR	BBS (pt) TUG (s)	NR	NR	NR	EXG vs. CPE Both groups ↑ BBS ↑ TUG
Pazzaglia et al. [34]	Italy	RCT	Subjects diagnosed with Parkinson’s Stage 2 through 3	EXG: 25 (72% male and 28% female) CPE: 26 (65.38% male and 34.62% female)	EXG: 72 ± 7.0 CPE: 70 ± 10.0	EXG: Virtual reality NIRVANA (BTS Spa, Garbagnate Milanese, Milan, Italy) CPE: Conventional balance and gait training	6	3	40	NR	BBS (pt) DGI (pt)	NR	NR	NR	EXG vs. CPE EXG ↑ BBS ↑ DGI
Pompeu et al. [42]	Brazil	RCT	Subjects diagnosed with Parkinson’s Stage 1 trough 3	EXG: 12 NR CPE: 12 NR	EXG: 60 to 85 CPE: 60 to 85	EXG: Nintendo Wii Fit CPE: Conventional balance training	7	2	30	NR	BBS (pt) -Unipedal Stance Test with eyes open -Unipedal Stance Test with eyes open, dual task condition -Unipedal Stance Test with eyes closed	MOCA (pt)	UPDRS-part II (pt)	NR	EXG vs. CPE Both groups ↑ MOCA ↔ Unipedal Stance Test with eyes open, dual task condition EXG ↑ BBS ↑ UPDRS-part II ↑ Unipedal Stance Test with eyes open ↑ Unipedal Stance Test with eyes closed
Ribas et al. [41]	Brazil	RCT	Subjects diagnosed with Parkinson’s Stage 1 through 3	EXG: 10 (40% male and 60% female) CPE: 10 (40% male and 60% female)	EXG: 61.70 ± 6.83 CPE: 60.20 ± 11.29	EXG: Nintendo Wii Fit CPE: Conventional balance training	12	2	30	NR	BBS (pt)	NR	NR	PDQ-39 (pt)	EXG vs. CPE EXG ↑ BBS ↑ PDQ-39
Shih et al. [35]	UK	RCT	Subjects diagnosed with Parkinson’s Stage 1 through 3	EXG: 10 (90% male and 10% female) CPE: 10 (70% male and 30% female)	EXG: 67.5 ± 9.96 CPE: 68.8 ± 9.67	EXG: Xbox Kinect balance CPE: Conventional balance training.	8	2	50	NR	Limits of stability -Reaction time -Movement velocity -Endpoint excursion -Directional control One-leg stance -Less affected with eyes open -More affected with eyes open -Less affected with eyes closed -More affected with eyes closed BBS (pt) TUG (s)	NR	NR	NR	EXG vs. CPE Both groups ↑ BBS ↑ TUG ↔ Movement velocity ↔ Less affected with eyes open ↔ More affected with eyes open ↔ More affected with eyes closed EXG ↑ Reaction time ↑ Endpoint excursion ↑ Directional control ↑ Less affected with eyes closed
Song et al [36]	Australia and New Zealand	RCT	Subjects diagnosed with Parkinson’s NR	EXG: 29 (52% male and 48% female) CPE: 25 (36% male and 64% female)	EXG: 68 (7) CPE: 65 (7)	EXG: open-source *Dance* *Revolution StepMania* CPE: Conventional balance training	12	3	15	NR	TUG (s) Functional gait assessments (pt) FES-I (pt)	MOCA (pt)	NR	NR	EXG vs. CPE Both groups ↔ Functional gait assessments ↔ FES-1 ↔ MOCA CPE ↑ TUG
Yang et al. [45]	UK	RCT	Subjects diagnosed with Parkinson’s Stage 2 through 3	EXG: 11 (63.64% male and 36.36% female) CPE: 12 (58.33% male and 41.67% female)	EXG: 72.5 ± 8.4 CPE: 75.4 ± 6.3	EXG: Touch screen computer with balance board CPE: Conventional balance training	6	2	50	NR	BBS (pt) TUG (s) DGI (pt)	NR	UPDRS-III (pt)	PDQ-39 (pt)	EXG vs. CPE Both groups ↔ UPDRS-III ↑ BBS ↑ TUG ↑ DGI ↑ PDQ-39

EXG: exergaming; CPE: conventional physical exercise; RCT: randomized controlled trial; NR: no reported; HRmax: heart rate maximum; RPE: rate of perceived exertion; POMA: Performance-Oriented Mobility Assessment; ABC Scale: Activities-specific Balance Confidence; BBS: Berg Balance Scale; DGI: dynamic gait index; MOCA: Montreal cognitive assessment; UPDRS-part II: Unified Parkinson’s Disease Rating Scale two; HRQoL: Health-related quality of life; UPDRS-part III: Unified Parkinson’s Disease Rating Scale three; PDQ-39: quality of life questionnaire for Parkinson’s Disease; TUG: Timed up and go; FES-I: Falls efficacy Scale International; pt: points. SPPB: short physical performance battery. ↑ improvement compared with the conventional physical exercise group (CPE); ↔ no change between groups.

**Table 4:** Exergaming vs. conventional physical exercise intervention on physical function, cognitive function, balance, fall risk, and health-related quality of life in older people with Parkinson’s disease.

Balance							
	n ^a^	Model Effect	ES (95%CI)	*p*	*I*^2^ (%)	Egger’s Test (*p*)	RW (%)
BBS (pts)	9, 9, 9, 306.	Random	0.90 (0.32 to 1.43)	**<0.01**	82.19	0.000	48.3 to 80.2
TUG (pts)	6, 6, 6, 170.	Random	0.16 (−0.51 to 0.84)	0.63	75.13	0.000	1.37 to 4.67
**Mobility and fall risk**							
DGI (pts)	3, 3, 3, 134.	Fixed	0.77 (0.42 to 1.11)	**<0.01**	**<0.01**	**0.61**	20.14 to 24.82
FES-I (pts)	3, 3, 3, 107.	Random	1.57 (−0.91 to 4.06)	0.21	96.09	0.00	0.98 to 3.36
**Physical and cognitive function**							
UPDRS-Part III (pts)	3, 3, 3, 92.	Random	−0.21 (−1.52 to 1.09)	0.75	89.41	0.000	−0.47 to 2.11
MOCA (pts)	3, 3, 3, 103.	Fixed	0.30 (−0.07 to 0.68)	0.11	**14.56**	**0.31**	4.83 to 8.10
**Health-related quality of life**							
PDQ-39 (pts)	4, 4, 4, 109.	Fixed	0.52 (0.14 to 0.90)	**<0.001**	**48.15**	**0.12**	13.25 to 14.25

Bolded p-values mean significant improvement (*p* < 0.05) in the experimental group after the exergaming intervention compared to conventional physical exercise group. **^a^** Data represent the number of studies included in the analysis, followed by the number of experimental (exergaming) and control (conventional physical exercise) groups, and the total number of participants considered in the meta-analysis. BBS: Berg Balance Scale; TUG: Timed Up-and-Go; DGI: Dynamic Gait Index; FES-I: Falls Efficacy Scale-International; UPDRS-part III: Unified Parkinson’s Disease Rating Scale three; MOCA: Montreal Cognitive Assessment; PDQ-39: quality of life questionnaire for Parkinson’s Disease.

**Table 5:** Results of the multivariate random-effect meta-regression for exergaming training variables, to predict exergaming training in effects on balance with BBS in older people with Parkinson’s disease.

Covariate	Coefficient	95% Cl	Z	*p*	R^2^
BBS (points) (n = 9)					
Intercept	−3.22	−6.87 to 0.42	−1.73	0.08	0.68
Weeks	0.21	0.11 to 0.31	4.10	**<0.01**	**0.80**
Frequency of training	0.14	−0.40 to 0.69	0.52	0.60	−0.29
Minutes per session	0.02	−0.03 to 0.08	0.71	0.47	−0.13
Total sessions	0.03	0.007 to 0.05	2.58	**0.01**	**0.49**

Bolded *p*-values indicate statistically significant effects (*p* < 0.05). BBS: Berg Balance Scale.

**Table 6:** GRADE assessment for the certainty of evidence.

Certainty Assessment							Number of Patients		Effect		Certainty	Importance
Number of Studies	Study Design	Risk of Bias	Inconsistency	Indirect Evidence	Vagueness	Other Considerations	[Intervention]	[Comparison]	Relative (95% CI)	Absolute (95% CI)		
**Balance**												
9	randomized trials	serious ^to^	it is not serious	it is not serious	it is not serious	none	148/306 (48.4%)	158/306 (51.6%)	not estimable		⨁⨁⨁ ◯ Moderate ^to^	IMPORTANT
**Mobility and fall risk**												
3	randomized trials	serious ^to^	it is not serious	it is not serious	it is not serious	none	65/134 (48.5%)	69/134 (51.5%)	not estimable		⨁⨁⨁ ◯ Moderate ^to^	IMPORTANT
**Physical function**												
3	randomized trials	serious ^to^	it is not serious	it is not serious	it is not serious	none	45/92 (48.9%)	47/92 (51.1%)	not estimable		⨁⨁⨁ ◯ Moderate ^to^	IMPORTANT
**Cognitive function**												
3	randomized trials	serious ^to^	it is not serious	it is not serious	it is not serious	none	55/103 (53.4%)	48/103 (46.6%)	not estimable		⨁⨁⨁ ◯ Moderate ^to^	IMPORTANT
**Health-Related Quality of Life**												
4	randomized trials	serious ^to^	it is not serious	it is not serious	it is not serious	none	64/109 (58.7%)	45/109 (41.3%)	not estimable		⨁⨁⨁ ◯ Moderate ^to^	IMPORTANT

Bolded text is used to highlight the outcome domains assessed in the GRADE analysis. ⨁⨁⨁◯ represents the overall certainty of evidence according to the GRADE approach, where ⨁⨁⨁◯ = moderate certainty. ^to^ indicates a range of severity judgments (e.g., from serious to very serious risk of bias).

## Data Availability

The datasets generated and/or analyzed during the current research are available from the corresponding author upon reasonable request.
